# Microbial communities from distinct *Vitis* species shape volatile profiles of fermenting juices while preserving varietal typicity

**DOI:** 10.3389/ffunb.2025.1643880

**Published:** 2025-09-18

**Authors:** María Laura Raymond Eder, Laura Fariña, Francisco Carrau, Alberto Luis Rosa

**Affiliations:** ^1^ Laboratorio de Genética y Biología Celular y Molecular, Departamento de Farmacología Otto Orsingher, Facultad de Ciencias Químicas, Universidad Nacional de Córdoba, Córdoba, Argentina; ^2^ Departamento de Agroalimentos, Facultad de Ciencias Agropecuarias, Universidad Nacional de Córdoba, Córdoba, Argentina; ^3^ Área Enología y Biotecnología de Fermentaciones, Facultad de Química, Universidad de la República, Montevideo, Uruguay; ^4^ Laboratorio de Biotecnología de Aromas, Facultad de Química, Universidad de la República, Montevideo, Uruguay; ^5^ Instituto de Farmacología Experimental de Córdoba (IFEC-CONICET), Córdoba, Argentina

**Keywords:** *terroir*, non-*Saccharomyces*, volatile profile, fermentation, Isabella

## Abstract

Spontaneously fermenting grape juices represent complex ecosystems resulting from the dynamic interaction between the unique characteristics of a grape varietal and its indigenous associated microbiota. The extent to which specific grape variety volatile compounds versus microbially derived ones shape wine identity remains incompletely understood. In this work, we explored this issue by characterizing the volatile compound profiles at early stages of fermentation of the highly aromatic Isabella (*V. labrusca* L.) grape juice, conducted by native microbial communities prepared from either Isabella (homologous fermentation) or Malbec (*V. vinifera* L., heterologous fermentation) grapes. Results revealed that microbial starters derived from *V. labrusca* L. and *V. vinifera* L. markedly influenced the volatile profiles of the resulting fermented Isabella grape juices. Joint analysis of volatile profiles from Malbec and Isabella juices fermented with the same set of *Vitis*-specific microbial communities showed that, despite the strong influence of the microbial consortia, the fermented juices retained traits consistent with their original grape varietal identity. Characterization and identification of cultivable yeast species in these homologous and heterologous fermentations of Isabella grape juice showed *H. uvarum*, *H. opuntiae*, and *S. bacillaris* as dominant species in Malbec and Isabella microbial ecosystems. Our results highlight the potential of this innovative experimental approach to examine the relative roles of microbial communities and grape varietals in shaping wine identity. Moreover, they show that different *Vitis*-specific microbiota can distinctly influence the volatile profiles of a fermenting grape juice without altering its varietal identity.

## 1 Introduction

Wine fermentation is a complex process where the interplay between grape juice and its associated microbial populations, derived from both the vineyard and winery environments, shapes the chemical and sensory profiles of the resulting wine (Belda et al., 2017; de Celis et al., 2022; García-Izquierdo et al., 2024). Yeasts are particularly important in alcoholic fermentation, where they convert sugars into alcohol and carbon dioxide, while also producing various secondary metabolites that contribute to the wine’s flavor and aroma (Maicas and Mateo, 2023; Wang et al., 2023). The indigenous yeast communities that develop during spontaneous grape must fermentations are shaped by annual environmental conditions in the vineyard, agricultural practices, intrinsic factors of the grapevine, —including the physicochemical properties of the grape must, — and winemaking techniques (Varela and Borneman, 2016; Griggs et al., 2021; Liszkowska and Berlowska, 2021; de Celis et al., 2022; Maicas and Mateo, 2023). A consistent pattern of species emerges in the yeast population: non-*Saccharomyces* yeasts dominate the initial phase of fermentation, while *Saccharomyces cerevisiae* becomes the predominant species as fermentation progresses (Bezerra-Bussoli et al., 2013; Drumonde-Neves et al., 2016; Cilião Filho et al., 2017; Raymond Eder et al., 2017, 2018; Borren and Tian, 2021; de Celis et al., 2022; Torres-Guardado et al., 2022; Maicas and Mateo, 2023). Non-*Saccharomyces* yeasts, which are predominant on grape skins, are introduced into the must during grape crushing and play a significant role in the early fermentation stages, producing various secondary metabolites that strongly impact the organoleptic characteristics of wine (Kamilari et al., 2021; Morata et al., 2021; Tufariello et al., 2021; Romano et al., 2022; Torres-Guardado et al., 2022; Maicas and Mateo, 2023; Wang et al., 2023).

Understanding the dynamics of indigenous microbial communities of fermenting grape juices and musts is a central focus in enology, given its significance for both scientific research and industrial applications (Padilla et al., 2016; Varela, 2016; Griggs et al., 2021; de Celis et al., 2022; Maicas and Mateo, 2023; García-Izquierdo et al., 2024). An increasing number of studies point to a relationship between the grape microbiome and *terroir*, which includes factors such as soil composition, climate, and annual precipitation, suggesting that specific microbial populations may contribute to the regional identity of wines (Pretorius, 2017, 2020; Liu et al., 2020; Griggs et al., 2021; Kamilari et al., 2021; Zhang et al., 2023a). Moreover, it has been suggested that particular grape varieties harbor microbiomes involving specific yeast strains or species, influencing the unique characteristics of fermented grape juices (Baffi et al., 2011; Bezerra-Bussoli et al., 2013; Lederer et al., 2013; Raymond Eder et al., 2017, 2018, 2025; Raymond Eder and Rosa, 2019). We have recently suggested that *Vitis*-specific microbial communities play a critical role in shaping the identity of grape juice fermentations (Raymond Eder et al., 2025). However, it remains unclear how strongly a grape varietal–specific indigenous microbial community influences the final organoleptic properties of a given wine, how *terroir*-associated microbial signatures contribute to the wine’s identity and profile, and to what extent alternative microbiotas—such as those from different vintages or experimentally introduced from other *Vitis* species (Raymond Eder et al., 2025)—can alter the fermentation profile of a given grape varietal.

Although the OIV (International Organization of Vine and Wine) primarily recognizes *Vitis vinifera* L. as the species designated for vinification, other *Vitis* species and their hybrids are also used in winemaking, particularly in non-European countries (De la Fuente Lloreda, 2018). These non-*vinifera Vitis* species represent underexplored microbial ecosystems in enology and may serve as a potential source of yeasts with unique fermentative properties of both academic and industrial relevance (Drumonde-Neves et al., 2016; Raymond Eder et al., 2017, 2018; Raymond Eder and Rosa, 2019). Moreover, we have recently proposed that they can serve as powerful tools for studying the impact of indigenous microbiota on the aromatic profiles of fermenting grape juices (Raymond Eder et al., 2025). Building on the use of this innovative experimental system, in this study we investigate the impact of the indigenous microbiota of Malbec grapes—a conventional *V. vinifera* L. varietal used in winemaking—versus the native microbiota of Isabella grapes (*V. labrusca* L.), on the volatile profile at early stages of fermentation of Isabella grape juices.

## 2 Materials and methods

### 2.1 Grape juice fermentations

**Figure 1:**
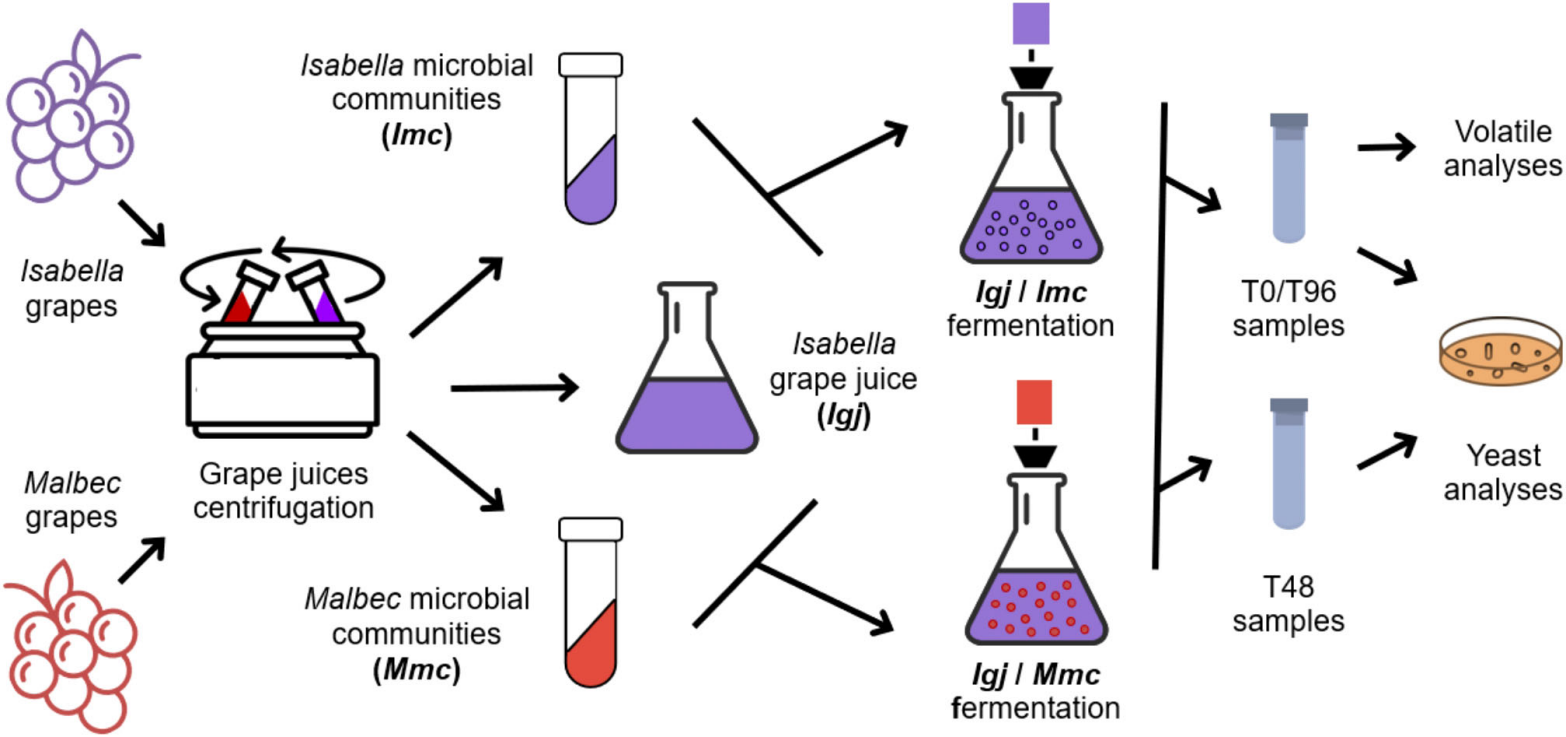
Experimental design flow-chart. 10 kg of Isabella and Malbec grapes were crushed, filtered, and the juices were centrifuged (5000 rpm, 10 min). The supernatant Isabella grape juice (*Igj*) was divided into 380 ml aliquots and pasteurized (60°C, 30 min). The Isabella (*Imc*) and Malbec (*Mmc*) sediments were resuspended in pasteurized Isabella juice and used to inoculate the pasteurized *Igj*. Triplicate fermentations were conducted for each *Igj/Imc* and *Igj/Mmc* at 25°C without agitation in Erlenmeyer flasks with air locks. Samples were taken every 24h from T0-T144. Appropriate dilutions of T0, T48, and T96 samples were plated and after incubation for 4 days at 25°C, yeast colonies were counted, isolated and identified. At T0 and T96 volatile compound levels (µg/l) were assessed by GC-MS.

Grapes from Isabella (*Vitis labrusca* L.) and Malbec (*Vitis vinifera* L.) were harvested from two closely located, small vineyards (i.e., ~1.5 Ha each), with intermixed rows of both varietals (Raymond Eder et al., 2018), in Colonia Caroya, Argentina, during the 2021 vintage. Isabella and Malbec grape juice supernatants (~3.5 l each), collected after centrifugation of filtered musts (5000 rpm for 10 min at 20°C), were pasteurized (60°C, 30 min) (Malletroit et al., 1991; Englezos et al., 2018; Raymond Eder et al., 2025). The corresponding Isabella (*I*) and Malbec (*M*) sedimented fractions, which contain their native microbial communities (i.e., *Imc* and *Mmc*, respectively) were each suspended into 400 ml of pasteurized Isabella grape juice (*Igj*) to reconstitute homologous (*Igj*/*Imc*) or heterologous (*Igj*/*Mmc*) fermenting ecosystems (Raymond Eder et al., 2025). Fermentations were performed in triplicate for each condition (*Igj*/*Imc* and *Igj*/*Mmc*) in 500 ml Erlenmeyer flasks, sealed with air locks, without agitation, at 25°C. Aliquots were collected from 0 to 96 hours (T0 to T96) for volatile, physicochemical, and microbial analyses, as previously described (Raymond Eder et al., 2025). **Figure 1** presents a schematic overview of the experimental design.

### 2.2 Volatile compounds identification and quantification

Volatile compounds were extracted by HS-SPME-GC-MS using an automatic injector AOAC-6000 Shimadzu, according to the methodology exposed by Garde-Cerdán et al. (2018). GC-MS analyses were conducted using a Shimadzu GC-20 plus gas chromatograph coupled to a Shimadzu QP 2020 mass spectrometer with a DW-Wax 30 (Agilent Technologies J&W, Santa Clara, CA, USA) bonded fused silica capillary column, coated with poly(ethylene glycol). The experimental conditions were performed according to Dellacassa et al. (2017) as follows: column temperature, 40°C for 8 min, rising to 180°C at 3°C/min, then to 230°C at 20°C/min; injector temperature, 250°C; detector temperature, 250°C; injection mode: splitless (2 min); carrier gas, hydrogen, 30 kPa. GC-MS instrumental procedures, using an internal standard (1‐heptanol), were applied for quantitative purposes (Dellacassa et al., 2017). Volatile compounds were identified by comparison of Kovats indices (KI, **Table 1**; **Supplementary Table S1**).

**Table 1:** Volatile compounds in *Igj* at T0 and in *Igj/Imc* and *Igj/Mmc* at T96.

Compounds	KI	*Igj*			*Igj/Imc*			*Igj/Mmc*		
		Mean	SD	Group	Mean	SD	Group	Mean	SD	Group
Higher alcohols										
Isoamyl alcohol	1221	13,79	0,86	a	198,12	104,82	b	93,56	31,79	b
3-etoxy-1-propanol	1378	0,00	0,00		0,53	0,22		0,67	0,59	
2-Ethyl-1-hexanol	1490	1,07	0,22		0,69	0,30		0,43	0,17	
2-nonanol	1530	0,00	0,00	a	3,84	1,46	b	5,82	0,60	b
1-octanol	1566	1,08	0,17		1,26	0,58		0,74	0,21	
2,3-butanediol	1590	0,27	0,01		1,28	1,84		0,99	0,62	
1-nonanol	1694	4,58	1,43		6,21	2,50		3,54	0,97	
Phenethyl alcohol	1918	14,91	2,86	a	77,48	14,20	c	44,45	6,46	b
*Total higher alcohols*		35,69	3,81	a	289,40	119,37	b	150,19	38,06	ab
Acetate esters										
Isoamyl acetate	1125	4,57	0,59	a	81,37	19,72	b	102,78	21,15	b
Hexyl acetate	1271	10,77	3,54		17,05	3,93		19,72	17,07	
(E)-3-Hexenyl acetate	1306	0,72	0,11	b	0,28	0,08	a	0,23	0,07	a
(Z)-3-Hexenyl acetate	1314	0,60	0,03		0,59	0,17		0,81	0,21	
Heptyl acetate	1382	1,93	0,47	b	0,62	0,20	a	0,94	0,30	a
Ethyl phenylacetate	1823	1,93	0,47	a	4,50	0,48	b	2,12	0,34	a
Phenethyl acetate	1822	3,10	0,87	a	56,49	9,57	b	20,76	6,43	a
*Total acetate esters*		23,61	2,24	a	160,91	33,05	b	147,36	27,83	b
Ethyl esters										
Ethyl 2-butenoate	1158	11,09	0,75	b	5,69	1,47	a	4,44	0,65	a
Ethyl hexanoate	1237	9,58	0,11		24,01	17,77		11,14	2,35	
Ethyl heptanoate	1323	0,46	0,04		1,36	0,97		0,78	0,25	
Ethyl lactate	1353	0,00	0,00	a	5,15	1,54	b	7,05	2,11	b
Ethyl 2-hexenoate	1360	8,14	0,68	b	3,45	0,87	a	2,95	0,33	a
Ethyl octanoate	1436	2,43	0,23	a	88,84	4,79	c	47,74	9,89	b
Ethyl 3-hydroxybutyrate	1530	0,85	0,06		0,62	0,10		0,62	0,24	
Ethyl nonanoate	1534	0,59	0,24	a	8,10	3,42	b	3,67	1,26	ab
Ethyl decanoate	1684	0,77	0,32	a	141,41	8,83	c	65,11	29,10	b
Ethyl dodecanoate	1822	0,16	0,08		119,10	89,29		43,69	21,73	
Ethyl hexadecanoate	2270	0,00	0,00	a	7,37	3,44	b	3,11	1,11	ab
*Total ethyl esters*		34,06	2,44	a	405,09	117,50	b	190,30	62,85	ab
C6 compounds										
Hexanol	1368	46,64	3,01		38,50	12,00		30,73	4,65	
(E)-3-hexen-1-ol	1372	0,67	0,00		0,54	0,23		0,44	0,07	
(Z)-3-hexen-1-ol	1388	0,86	0,10	b	0,59	0,12	ab	0,33	0,05	a
*Total C6 compounds*		48,17	2,91	a	39,64	12,34	a	31,50	4,77	a
Terpenes										
limonene	1190	0,66	0,24		1,17	0,49		1,05	0,23	
Linalol	1558	1,20	0,35		2,03	0,64		1,22	0,18	
4-terpineol	1630	0,59	0,11		0,79	0,24		0,68	0,11	
alpha terpineol	1718	2,30	0,65		3,68	1,58		2,31	0,63	
Nerol	1810	0,14	0,08		0,98	0,52		0,49	0,04	
(E,E)-Farnesol	2350	0,00	0,00		0,77	0,53		0,22	0,10	
*Total terpens*		4,89	1,43	a	9,42	3,94	a	5,96	1,05	a
Miscelaneous										
2-heptanone	1180	0,00	0,00	a	0,83	0,53	ab	1,57	0,56	b
Acetoin	1266	0,00	0,00		3,03	2,15		2,02	1,24	
2-nonanone	1395	0,28	0,03	a	3,44	1,72	a	12,08	3,08	b
Benzaldehyde	1540	6,98	2,59	b	1,01	0,36	a	0,98	0,45	a
Acetophenone	1670	0,40	0,38		1,15	0,23		1,02	0,38	
Methyl salicylate	1735	0,58	0,10		0,97	0,19		1,06	0,63	
(Z)-Methyl cinnamate	2080	0,64	0,29		1,19	0,58		0,48	0,11	
Methyl antranilate	2255	0,06	0,00	a	0,25	0,03	b	0,05	0,01	a
*Total miscelaneous*		8,93	3,33	a	11,87	3,37	ab	19,25	2,29	b
*Total volatile compounds*		155,35	16,16	a	916,34	289,56	b	544,55	136,85	ab

*Kovats Index. Identities confirmed by comparing mass spectra and linear retention indices with those of authentic standards supplied by Aldrich (Milwaukee, WI, USA) and Fluka (Buchs, Switzerland), or reported in the literature. Values (μg/l) correspond to the mean of two (*Igj* T0) or three (*Igj/Imc* and *Igj/Mmc*) replicas ± SD. Values with a common letter are not significantly different (p >0.05).

An ANOVA was conducted on the chemical and volatile compounds data obtained for the different treatments and replicas. Principal Component Analysis (PCA) was performed using InfoStat (InfoStat, FCA, Universidad Nacional de Córdoba, Argentina) to differentiate between samples and identify contributing compounds. A Hierarchical Cluster Analysis (HCA) was performed using Euclidean distances calculated from the average volatile profiles of each fermenting condition. Clustering was performed using the complete linkage method. The resulting dendrogram was constructed using base R functions (dist and hclust) and visualized with the *dendextend* package for enhanced customization.

### 2.3 Yeast identification from Isabella fermentations

Yeasts were isolated from Isabella homologous and heterologous fermentations at different time points (T0, T48, and T96 h) by plating pooled samples on YPD-Cm [yeast extract 1.0% (w/v), peptone 2.0% (w/v), glucose 2.0% (w/v), agar 2.0% (w/v), chloramphenicol 10 µg/ml] and YPD-Cm-Cx agar [yeast extract 1% (w/v), peptone 2% (w/v), glucose 2% (w/v), agar 2% (w/v), chloramphenicol 10 µg/ml, cycloheximide 0.5 µg/ml] to estimate total and non-*Saccharomyces* populations, respectively (Raymond Eder et al., 2025). Predominant yeast species were randomly selected from high-dilution YPD-Cm plates using a grid-based method to ensure unbiased isolation (Raymond Eder et al., 2017). Colonies were purified, stored in glycerol stocks, and subsequently identified by PCR-RFLP and/or sequencing of the 5.8S-ITS rDNA region. Restriction profiles were generated using *Hinf I* and *CfoI*, and representative isolates were confirmed by Sanger sequencing, with species assignment based on ≥99% identity to reference sequences in NCBI BLAST. Identified yeast strains were deposited in GenBank (NCBI) under the accession numbers OP584257, OP584258, OP584259, OP584260, OP584261, OP584262, OP584263, and OP584265.

## 3 Results

### 3.1 Physicochemical and volatile analyses of Isabella grape juice

High nitrogen compounds levels, such as α Amino (171.1 ± 4.3 mg/l), NH_4_^+^ (77.8 ± 4.2 mg/l), and yeast assimilable nitrogen-YAN- (235.5 ± 7.8 mg/l) were found in the *Igj*, as well as a total acidity value of 2.9 ± 0.0 g/l, a density of 1.083 ± 0.001 g/ml, and 19.1 ± 0.4°Brix. Volatile compounds in the *Igj* at T0 were analyzed using GC-MS (**Table 1**; **Supplementary Table S1**). The detected compounds exclude bound volatiles and potential artifacts arising from enzymatic treatments used for their release, thus accurately reflecting the aroma-active profile of a fresh grape juice (Raymond Eder et al., 2025). The recognized profile of *Igj* is distinguished by the presence of the two correlated aroma terpenes α-terpineol/linalool, a characteristic marker of the Isabella varietal, along with a high proportion of alcohols, particularly hexanol, phenethyl alcohol, isoamyl alcohol, and 1-nonanol, followed by some ethyl esters of medium chain fatty acids (**Table 1**; **Supplementary Table S1**). Ethyl 3-hydroxybutyrate and methyl anthranilate, previously identified as free volatile aroma compounds in Isabella grape samples (Ghaste et al., 2015), were also recognized in the analyzed grape juices. In particular, methyl anthranilate is associated with the perception of foxiness in *V. labrusca* L. grapes (Raymond Eder and Rosa, 2019).

### 3.2 Isabella grape juice fermentations

Indigenous microbial communities from Isabella (*Imc*) and Malbec (*Mmc*) grapes were prepared as indicated (Raymond Eder et al., 2025) (see also **Figure 1**). As recently reported, the centrifugation process enabled effective collection of the cultivable yeast community; additionally, the pasteurized grape juices showed no detectable presence of cultivable yeasts (i.e., <10 CFU/ml) (Raymond Eder et al., 2025). The mild pasteurization applied (i.e., 60°C for 30 minutes) is much less intense than other processes studied (i.e., 81.5 ± 0.5°C for 450 min) (Malletroit et al., 1991), preserving the sensory profile of the grape juice (Malletroit et al., 1991; Raymond Eder et al., 2025).

**Figure 2:**
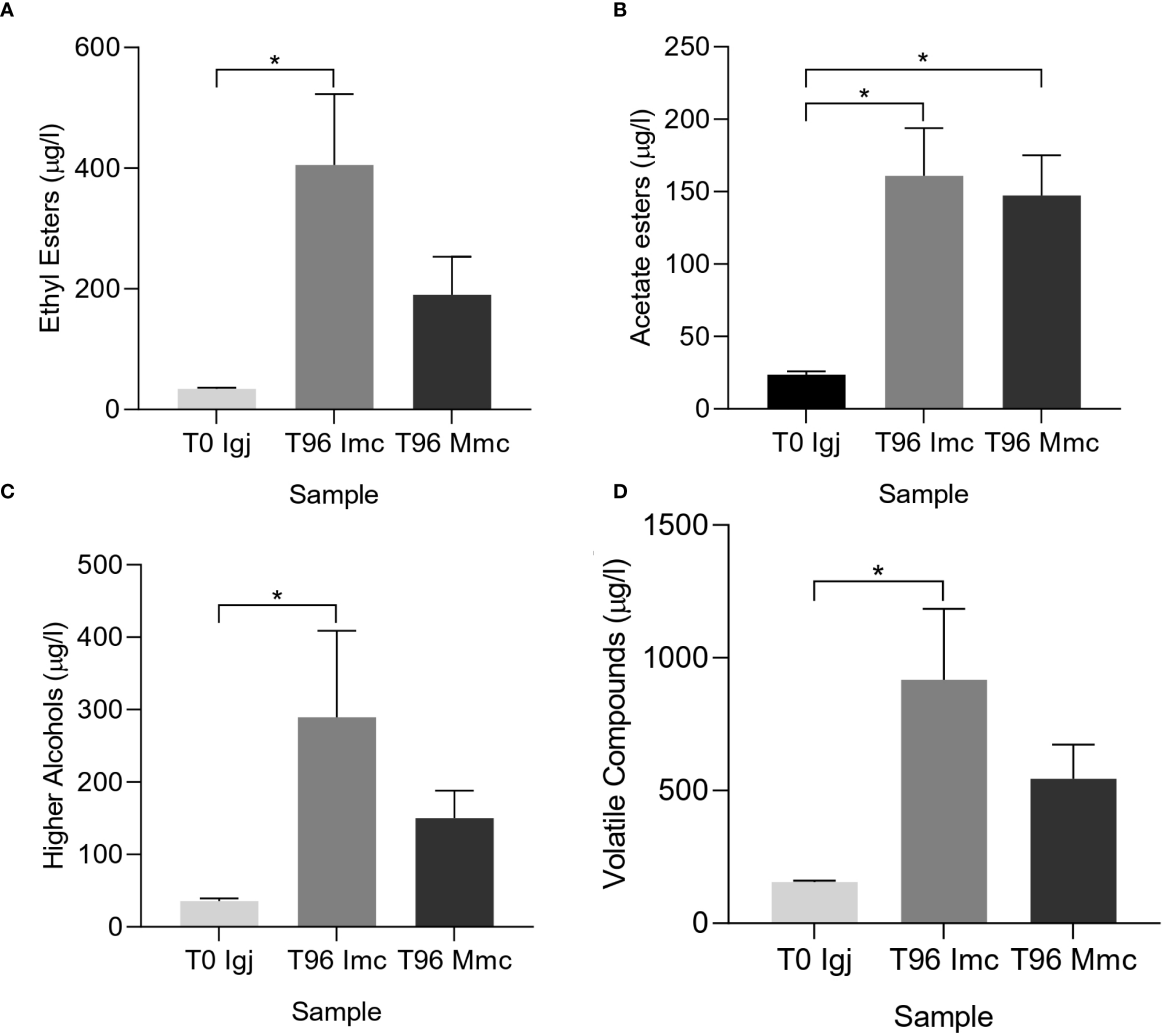
Concentration of volatile compounds (µg/l) determined by GC-MS in Isabella grape juice (T0) and after 96 h of fermentation using *Imc* (T96 Imc) and *Mmc* (T96 Mmc) as inocula. Total ethyl ester **(A)**, acetate ester **(B)**, higher alcohol **(C)**, and volatile **(D)** compound concentrations (see **Table 1**). Values are means ± SD (**Table 1**; **Supplementary Table S1**). Significant differences (p <0.05) are marked with an asterisk *.

The pasteurized *Igj* was inoculated with *Imc* and *Mmc* and their effect on the volatile profiles of fermenting *Igj* were evaluated from both homologous (*Igj*/*Imc*) and heterologous (*Igj*/*Mmc*) fermentations. After four days of fermentation (i.e., T96), a total of forty-three compounds, including acetates, alcohols, esters, and terpenes, were identified and quantified in *Igj* (**Table 1**; **Supplementary Table S1**). Significant differences in the overall volatile compound concentrations were observed between *Igj*/*Imc* T0 and T96 fermentations (**Figure 2**; **Table 1**; **Supplementary Table S1**). The varietal compounds (E)-3-hexenyl acetate, heptyl acetate, ethyl 2-butenoate, ethyl 2-hexenoate, identified in *Igj* T0 samples, were found in lower concentrations at T96. At this fermenting time, the most abundant compounds in both conditions were isoamyl alcohol, phenethyl alcohol, isoamyl acetate, phenethyl acetate, ethyl octanoate, ethyl decanoate, and ethyl dodecanoate (**Table 1**). The compounds ethyl lactate, ethyl hexadecanoate, 2-nonanol, (E,E)-farnesol, 2-heptanone, and acetoin were identified only at T96. The terpenic and C6 compounds identified in Isabella grape juices showed no significant differences between fermenting times (i.e., T0 and T96; **Figure 2**). Significant differences were found for ethyl octanoate, ethyl decanoate, phenethyl alcohol, phenethyl acetate, and ethyl phenylacetate, which were quantified in higher concentrations in the homologous fermentations (**Table 1**; **Supplementary Table S1**; **Figure 2**).

**Figure 3:**
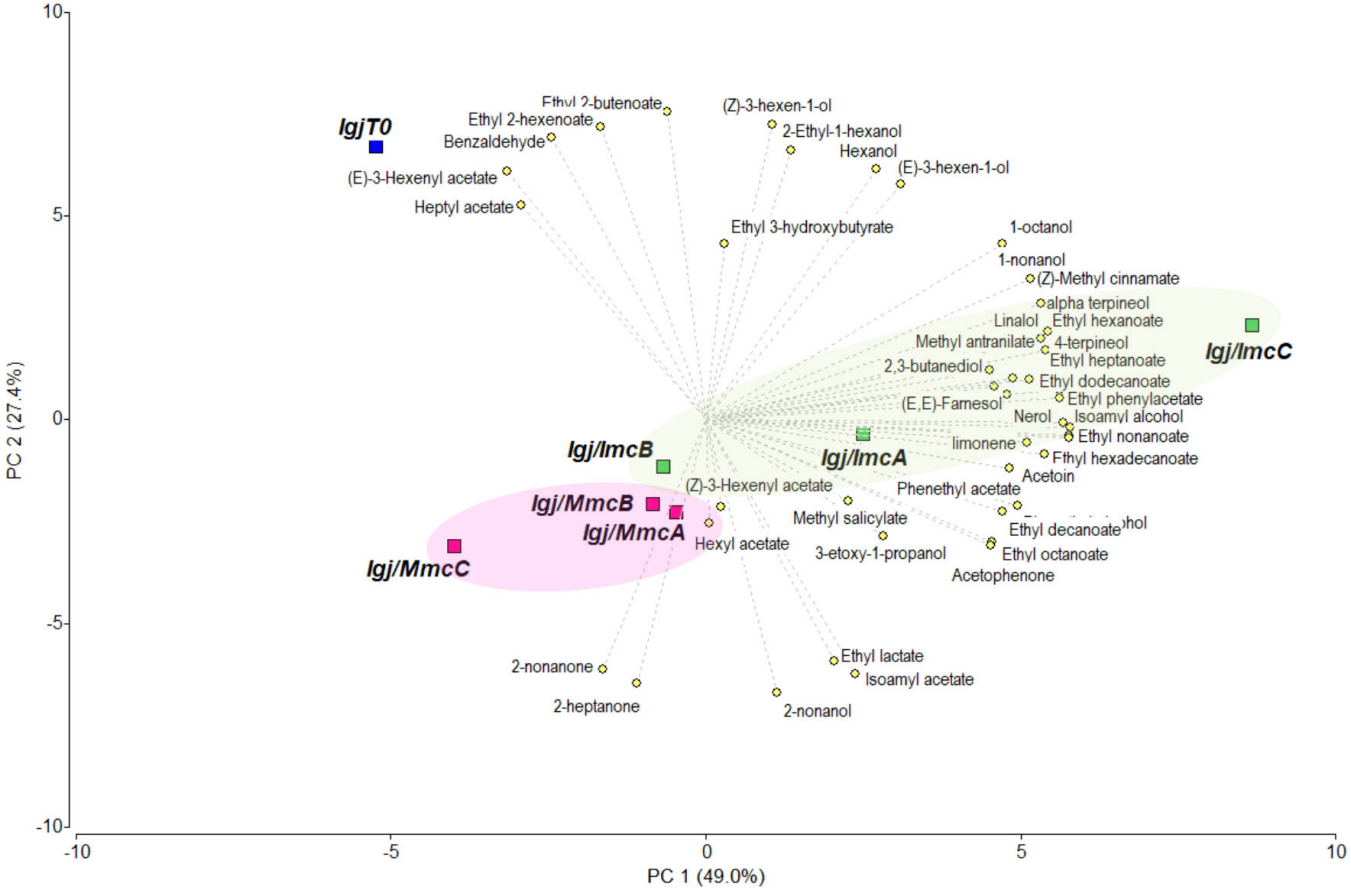
Principal Component Analysis (PCA) performed on the concentration of the volatile compounds identified in the Isabella grape juice at T0 and the *Igj/Imc* and *Igj/Mmc* fermenting conditions at T96 (**Table 1**). Representation of the volatile compounds, the Isabella grape juice at T0 (i.e., *IgjT0*), and three replicas (i.e., A, B, C) of each fermenting condition using *Imc* (i.e., *Igj/ImcA, Igj/ImcB, Igj/ImcC*) or *Mmc* (i.e., *Igj/MmcA, Igj/MmcB, Igj/MmcC*) at T96 in the two first components (PC1 and PC2).

The volatile compound profiles of fermented *Igj* using the alternative *Imc* and *Mmc* starters were further analyzed through Principal Component Analysis (PCA). **Figure 3** illustrates that PC1 accounted for 49.0% of the variance, while PC2 accounted for 27.4%. This analysis demonstrated a distinct separation between fermented (i.e., T96) and non-fermented (i.e., T0) grape juices. Moreover, among the fermented grape juices, the analysis revealed clustering based on the starter used for fermentation (**Figure 3**). In **Figure 3**, *Igj/Mmc* fermentations are located in the negative values of PC1, whereas *Igj/Imc* samples cluster together in the positive values of PC1, primarily associated with ethyl esters, which are volatile aroma compounds found in significantly higher quantities under this fermenting condition.

### 3.3 Impact of homologous and heterologous microbial starters on the volatile profiles of Malbec and Isabella grape juice

**Figure 4:**
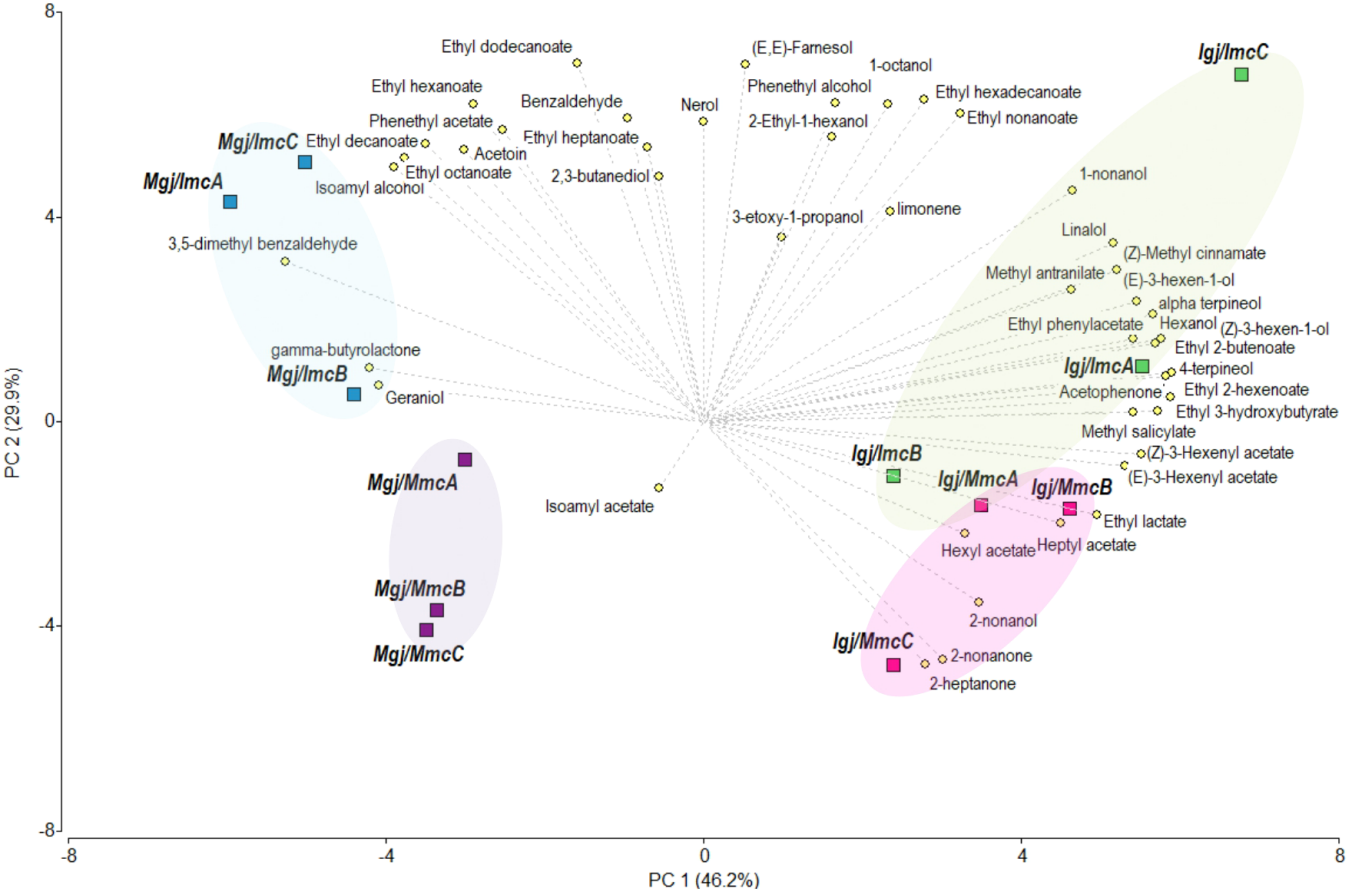
Principal Component Analysis (PCA) performed on the concentration of the volatile compounds identified in the *Igj/Imc* and *Igj/Mmc* fermenting conditions at T96 and the *Mgj/Mmc* and *Mgj/Imc* fermenting conditions at T96 reported in (Raymond Eder et al., 2025) (**Supplementary Table S2**). Representation of the volatile compounds and the three replicas (i.e., A, B, C) of each fermenting condition at T96 in the two first components (PC1 and PC2). Compounds that were not identified at T96 and compounds that did not increase their concentration from T0 to T96 were not considered in this analysis.

To evaluate the relative influence of the fermentation inoculum and the fermenting grape juice on volatile profiles, we conducted a PCA integrating the volatile profile data obtained in this study from *Igj*/*Imc* and *Igj*/*Mmc* fermentations at T96 with previously published data from Malbec grape juices (*Mgj*) (Raymond Eder et al., 2025), fermented using the identical *Imc* and *Mmc* inocula as in the present work. All Isabella and Malbec samples were prepared from grapes harvested in the same vintage and processed and fermented in parallel, which enables proper comparison of previous and current datasets (see Materials and Methods section) (Raymond Eder et al., 2025). **Figure 4** shows that PC1 counted for 46.2%, while PC2 for the 29.9% of the variance. The PC1 showed a separation between the grape juice used for fermentation, with *Igj* (this work) and *Mgj* (Raymond Eder et al., 2025) locating at positive and negative values of the graph, respectively. An ANOVA performed on the miscellaneous varietal compounds (i.e., acetophenone, methyl salicylate, 3,5-dimethyl benzaldehyde, (z)-methyl cinnamate, and methyl anthranilate; **Supplementary Table S2**), revealed significant differences among the homologous fermentations (i.e., *Igj/Imc* and *Mgj/Mmc*). However, similar concentrations of these compounds were found in Isabella and Malbec fermentations performed with the heterologous microbial communities (i.e., *Igj/Mmc* and *Mgj/Imc*; **Supplementary Table S2**), suggesting that these communities reduce the varietal variability in these fermentations. Regarding the impact of the microbial communities in the volatile profiles, a differentiation among the microbial communities, although to a limited extent in the *Igj* fermentations, is observed in the PC2. In general, the *Imc* replicas were positioned in positive values, while *Mmc* were positioned in negative values in the analysis. To further analyze these results, a hierarchical cluster analysis (HCA) based on Euclidean distances between the average volatile profiles of each fermenting condition was performed and plotted (**Supplementary Table S3**; **Supplementary Figure S1**). The resulting dendrogram shows that the volatile profiles of fermenting grape juices cluster primarily according to the microbial community used as inoculum. Specifically, samples inoculated with the Malbec microbial community (*Mmc*) cluster together (*Mgj*/*Mmc* and *Igj*/*Mmc*), regardless of grape variety, indicating a strong shaping effect of this community. In contrast, the two samples inoculated with the Isabella microbial community (*Imc*) are more distant from each other, with *Mgj*/*Imc* appearing as the most distinct condition overall. These results suggest that the microbial community has a dominant influence on the volatile profile, but the interaction with grape varieties also contributes to the observed variability.

A factorial ANOVA was performed in order to evaluate the impact of the analyzed factors (i.e., grape juice -either *Igj* or *Mgj*- and inoculum -either *Imc* or *Mmc*-) and their interactions in the volatile profile variabilities. This analysis revealed that there were no significant effects from the interactions between the two factors for any of the volatile compounds identified (**Supplementary Table S2**). The grape juice and inoculum, however, significantly impacted 30 and 16 volatile compounds, respectively. The compounds phenethyl alcohol, phenethyl acetate, ethyl nonanoate, ethyl decanoate, ethyl hexadecanoate, and (E,E)-farnesol were the only compounds significantly impacted only by the inoculum. Interestingly, phenethyl acetate, ethyl decanoate, and ethyl hexadecanoate were present in significantly higher concentrations in fermentations performed using *Imc* (**Supplementary Table S2**).

These analyses reveal that alternative microbial communities (i.e., *Imc* or *Mmc*) can shape the profile of volatile compounds in ways that allow differentiation between fermentation conditions, while the fermenting grape juices preserve the aromatic typicity and varietal identity of each cultivar (**Figure 4**).

### 3.4 Yeast communities in fermenting Isabella grape juices

**Figure 5:**
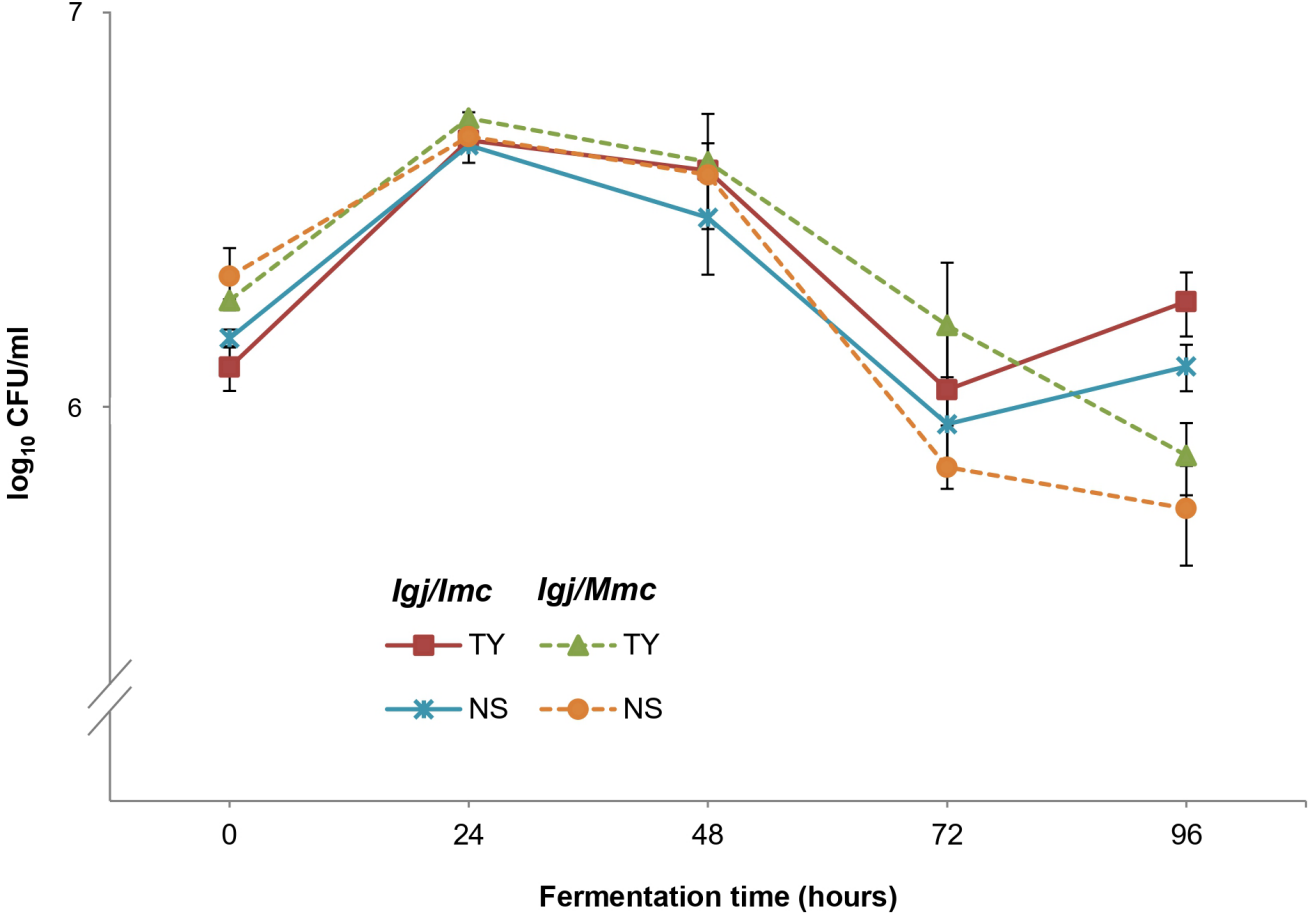
Population dynamics of non-*Saccharomyces* (NS) and total yeasts (TY) at initial stages of fermentations (0 to 96 h) of Isabella grape juice (*Igj*) using Isabella (*Imc*) or Malbec (*Mmc*) microbial communities as inocula. Each point represents log_10_ CFU/ml ± SD.

**Figure 6:**
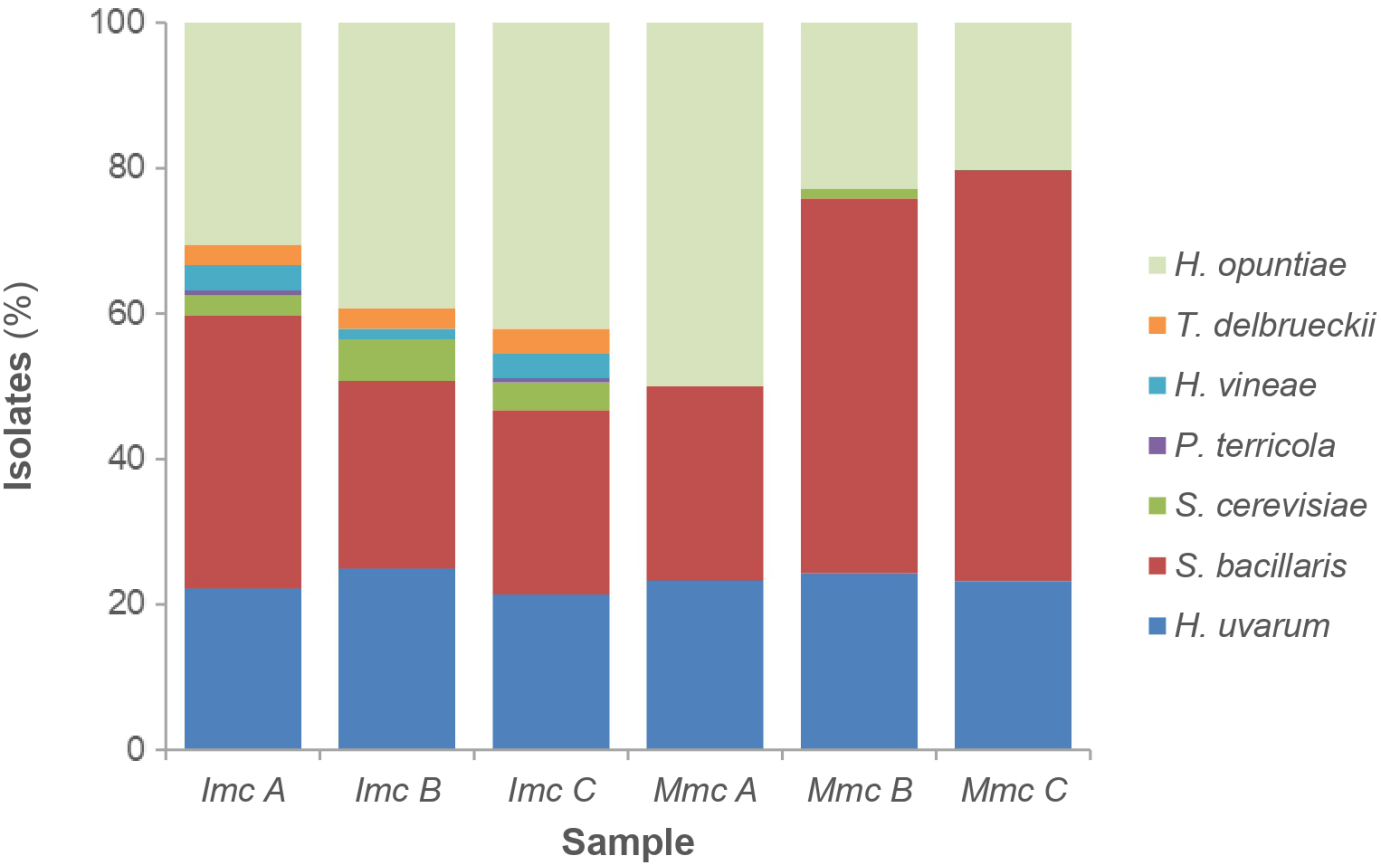
Main represented yeast species at T96 of the three replicas from each fermenting condition. Appropriate dilutions of replicas A, B, and C from *Imc* (left; columns 2–4) and *Mmc* (right; columns 6–8) conditions were plated individually, and percentages represent the relative contribution of the indicated yeast species.

Differential CFU/ml counts obtained from YPD-Cm and YPD-Cm-Cx agar plates (Raymond Eder et al., 2017, 2018) were used to assess the contributions of total yeasts and non-*Saccharomyces* yeasts, respectively. Results from this analysis revealed that both conditions (i.e., *Igj/Imc* and *Igj/Mmc*) started with similar counts (~1–2 x 10^6^ CFU/ml), with non-*Saccharomyces* species predominating during the early stages of fermentation (T0 to T96) (**Figure 5**; **Supplementary Figure S2**). *S. cerevisiae* started to become dominant at middle stages of fermentation (i.e., T144; not shown). Based on these results, we focused our analyses of non-*Saccharomyces* species present at early stages of fermentations (i.e., T0 to T96). A total of 140 isolates from *Igj/Imc* and *Igj/Mmc* were isolated and identified (**Supplementary Figure S2**). *Hanseniaspora opuntiae* and *Hanseniaspora uvarum* were the most common species at early stages of *Igj*/*Imc* and *Igj*/*Mmc* fermentations (**Supplementary Figure S2**), followed by *Starmerella bacillaris*. Similar results have been obtained when analyzing grape-associated indigenous yeast communities from this ecosystem (Raymond Eder et al., 2017, 2018, 2025; Raymond Eder and Rosa, 2019). Even if similarities were found among the main recognized yeast species in the homologous and heterologous fermenting samples (i.e., *Igj*/*Imc* and *Mgj*/*Imc*, respectively), differences in the relative proportion of these yeasts were observed at the analyzed times (**Figure 3**). Also, *Igj/Imc* fermentations at T96 revealed the presence of *Torulaspora delbrueckii*, *Hanseniaspora vineae*, and *Pichia terricola* as representative isolates (**Figure 6**). This greater yeast biodiversity, as well as changes in the relative contribution of the main yeast species identified in the fermentations, could help explain the variations observed in the evolving volatile profiles of *Igj*/*Imc* replicates (**Figure 3**).

## 4 Discussion

Varietal or primary wine flavors originate from the grape variety itself. In addition to the volatile compounds naturally present in grape juice, secondary flavors are developed through alcoholic fermentation via yeast metabolism, which produces higher alcohols, esters, and fatty acids (Carrau et al., 2020; Borren and Tian, 2021; Zilelidou and Nisiotou, 2021; Romano et al., 2022; Liang et al., 2023; Wang et al., 2023; Zhang et al., 2023a). In spontaneous fermentations, the complex native microbial community associated to grapes contributes to the final volatile profiles of wines (Padilla et al., 2016; Belda et al., 2017). Indigenous grape-associated yeasts species are particularly relevant contributors to the volatile profiles of fermenting juices at early times of fermentation (Jolly et al., 2014; Pretorius, 2017; Raymond Eder et al., 2025). In addition to yeast, lactic-acid and acetic-acid bacteria, even when present at low concentrations at early times of fermentations, can also contribute to the final volatile compositions of spontaneous fermentations (Barata et al., 2012; Andorrà et al., 2019; Shimizu et al., 2023; Raymond Eder et al., 2025).

We have recently demonstrated that microbial communities associated to different *Vitis* species condition the volatile profiles of early-stage Malbec grape juice fermentations (Raymond Eder et al., 2025). In this work, we have extended these studies to analyze the volatile profiles of Isabella grape juices fermented using homologous (Isabella; *V. labrusca* L.) or heterologous (i.e., Malbec; *V. vinifera* L.) native microbial communities. Our analyses consider the profiles of free volatile compounds in grape juices, excluding glycoside-bound volatiles (Raymond Eder et al., 2025). Although bound volatiles contribute to the full aromatic potential of a grape juice —upon enzymatic or fermentative release— the analyzed free fraction offers a representative expression of varietal identity at the juice stage. Moreover, this approach avoids potential artifacts introduced by exogenous enzymes, yields reproducible data under our standardized conditions, and reveals the aromatic identity of fresh varietal grape juice. This experimental strategy allowed us to perform a joint analysis of the volatile compound datasets obtained from homologous and heterologous fermentations of Malbec (Raymond Eder et al., 2025) and Isabella (this work) grape juices, conducted with the same starter microbial communities and grape juices samples prepared from grapes harvested in vintage of year 2021 (Raymond Eder et al., 2025). Taken together, the results presented in this work show that the microbiota shapes the volatile profiles of the fermented Malbec and Isabella juices, which nonetheless retain the identity of their original grape varietal.

It has been reported that Isabella, a varietal from the American-originated *Vitis labrusca* L., is rich in aroma compounds both qualitatively and quantitatively (Ghaste et al., 2015). Key alcohols and terpenes, including methyl anthranilate, β-phenyl ethanol, ethyl-3-hydroxybutyrate, ethyl-β-hydroxy hexanoate, furaneol, phenylacetaldehyde, tryptophol, and 2-hexenol, have been detected in Isabella grape juices and crushed grapes originating from Brazil and Italy, respectively (Ghaste et al., 2015; Gomez et al., 2024). Our analysis revealed that Isabella juice from grapes harvested in the geographic region of Córdoba (Argentina) has a substantial proportion of alcohols, esters, and terpenes, including the previously reported methyl anthranilate, β-phenyl ethanol, ethyl-3-hydroxybutyrate, and methyl salicylate (Ghaste et al., 2015; Gomez et al., 2024). Although different techniques of extraction of volatile compounds were used, Isabella juice from Córdoba was found to contain eleven ethyl esters, whereas only two (i.e., ethyl-butanoate and ethyl-metacrylate) and four (i.e., methyl salicylate, methyl anthranilate, methyl-β-hydroxybutyrate, ethyl-3-hydroxyhexanoate) ethyl esters were detected in the Brazilian and Italian studies, respectively (Ghaste et al., 2015; Gomez et al., 2024). Previous research on the volatile profiles of Isabella and Ives commercial wines also emphasized the prominent role of ethyl acetate and esters, contributing to their characteristic fruity aroma descriptors (Arcanjo et al., 2018). In the present study, isoamyl alcohol, phenethyl alcohol, isoamyl acetate, phenethyl acetate, ethyl octanoate, ethyl decanoate, and ethyl dodecanoate emerged as the dominant volatile compounds during the initial stages of fermentation. Interestingly, as it was observed in our recent study of homologous and heterologous fermentation of Malbec grape juice (Raymond Eder et al., 2025), differences were observed in the volatile profiles of the Isabella fermenting grape juice when using the alternative either Malbec or Isabella associated microbial communities as starters. Homologous fermentations (*Igj*/*Imc*) show higher concentrations of ethyl octanoate, ethyl decanoate, phenethyl alcohol, phenethyl acetate, and ethyl phenylacetate than the heterologous conditions (*Igj*/*Mmc*) (Raymond Eder et al., 2025).

The evolution of culturable yeast populations in homologous and heterologous Isabella grape juice fermentations was studied using culture-dependent methods. Although his strategy limits our study to the identification of only cultivable yeast species from the overall fungal, yeast, and bacterial biodiversity in the samples, it is known that cultivable non-*Saccharomyces* yeast species significantly contribute to aromatic profiles in early fermentation stages (Andorrà et al., 2010; Borren and Tian, 2021; Zilelidou and Nisiotou, 2021; Romano et al., 2022; de Celis et al., 2024). At T0, *Hanseniaspora* spp. (*H. opuntiae* and *H. uvarum*) and, to a lesser extent, *S. bacillaris*, predominated in the fermented juices, consistent with previous studies on yeast communities from this region’s spontaneously fermenting musts and reconstituted grape juices (Raymond Eder et al., 2017, 2018, 2025). As fermentation progressed, from T0 to T96, *Hanseniaspora* spp. dominated the fermentations in *Imc*, while *S. bacillaris* increased its contribution in *Mmc*. It has been recently reported that the dominating yeast species in a fermentation defines its performance and metabolite profile of the resulting wines (de Celis et al., 2024). In this sense, members of the *Hanseniaspora* genus have been reported to play a significant role in the production of volatile compounds in wine, particularly acetate esters, in a strain-dependent manner (Jolly et al., 2014; Martin et al., 2018). Specifically, *H. uvarum* significantly increased ethyl and isoamyl esters and reduced acetate ester concentrations in co-fermentations with *S. cerevisiae* in a strain-specific way (Tristezza et al., 2016; Hu et al., 2018b, 2018a; Zhang et al., 2023b). *H. opuntiae* has been shown to positively influence volatile profiles and sensory characteristics of fermentations, significantly increasing acetate esters, mainly 2-phenylethyl acetate, and ethyl esters levels (Hu et al., 2020; del Fresno et al., 2022, 2023; Filippousi et al., 2024). Moreover, fermentation studies where *Hanseniaspora* spp. were the dominating yeasts, were characterized by higher fusel alcohol acetates production (de Celis et al., 2024). (i.e., phenethyl alcohol). On the other hand, *S. bacillaris* has been reported to overproduce ethyl and other acetate esters, as well as terpenes (Andorrà et al., 2010; Englezos et al., 2015; Raymond Eder and Rosa, 2021). However, elevated levels of these compounds were not detected in the *Igj*/*Mmc* fermentation, where this yeast was dominant at T96. Four other non-*Saccharomyces* species previously recognized in this *terroir* (i.e., *Candida diversa*, *T. delbrueckii*, *H. vineae*, and *P. terricola*) were also isolated at these fermentations. Proposed signature *V. labrusca* L. non-*Saccharomyces* species (i.e., *Candida azymoides*, *Pichia cecembensis*, and *Candida californica*) (Drumonde-Neves et al., 2016; Raymond Eder et al., 2017, 2018) were not identified among the limited number of isolates characterized in this study. In addition to the contribution to the volatile profiles of the main yeast species, increased yeast diversity has been linked to greater flavor complexity (Carrau and Henschke, 2021). In this context, the minor yeast species present at lower abundances in *Igj*/*Imc* may have also contributed to the elevated concentrations of certain volatile compounds. *T. delbrueckii*, for instance, enhances succinic acid, linalool, acetate esters, medium chain fatty acids, and terpenes in aromatic grapes (Jolly et al., 2014; Varela and Borneman, 2016). Similarly, *P. terricola* has been reported to release precursors from grape juice, boosting free monoterpenes and norisoprenoids (Jolly et al., 2014). The apiculate yeast *H. vineae* has been reported to produce terpenes, sesquiterpenes, and high amounts of acetate esters, such as 2-phenylethyl acetate and ethyl acetate, which influence fermentations by producing flavors and increasing sensory complexity (Martin et al., 2018).

Our results show that microbial communities assembled in different *Vitis* species influence the volatile profiles of early-stage fermentations of different grape juices. Additional data from fermentations using other microbial communities and grape juice varietals would strengthen the conclusion that alternative yeast populations shape the volatile profiles of fermented juice while preserving grape varietal identity. Also, grape fermentations using pure cultures of selected non-*Saccharomyces* strains, and/or pools of selected strains that resemble native microbial communities, could further clarify the specific contributions of individual yeast species and strains to the final volatile profiles of fermented grape juices. Emerging evidence suggest that non-conventional *Vitis* ecosystems may carry unique yeast species or strains not found in *V. vinifera* L., presenting opportunities for the isolation of valuable *Saccharomyces* and non-*Saccharomyces* strains of potential relevance for the winemaking industry.

## Data Availability

The original contributions presented in the study are publicly available. This data can be found here: NCBI GenBank, accession OP584257, OP584258, OP584259, OP584260, OP584261, OP584262, OP584263, and OP584265.

## Supplementary material

Supplementary Figure 1 Dendrogram resulting from hierarchical cluster analysis based on Euclidean distances calculated from the average profiles of volatile compounds in each fermenting condition (i.e., *Igj/Imc*, *Igj/Mmc*, *Mgj/Mmc*, and *Mgj/Imc*) at 96 hours (**Supplementary Table S3**). Each point of the dendrogram represents the centroid of three biological replicates performed in the study.

Supplementary Figure 2 Main represented yeast species at the initial stages of fermentations (0 to 96 h) of Isabella grape juice using Isabella (*Imc*) or Malbec (*Mmc*) microbial communities as inocula. Percentages represent the relative contribution of the indicated yeast species among the colonies obtained at the indicated times of fermentation.
