# Supplementary figures and images for: Microbial communities from distinct Vitis species shape volatile profiles of fermenting juices while preserving varietal typicity
Source: Front Fungal Biol. 2025 Sep 18;6:1643880. doi: 10.3389/ffunb.2025.1643880 (PMC12489950; doi:10.3389/ffunb.2025.1643880)

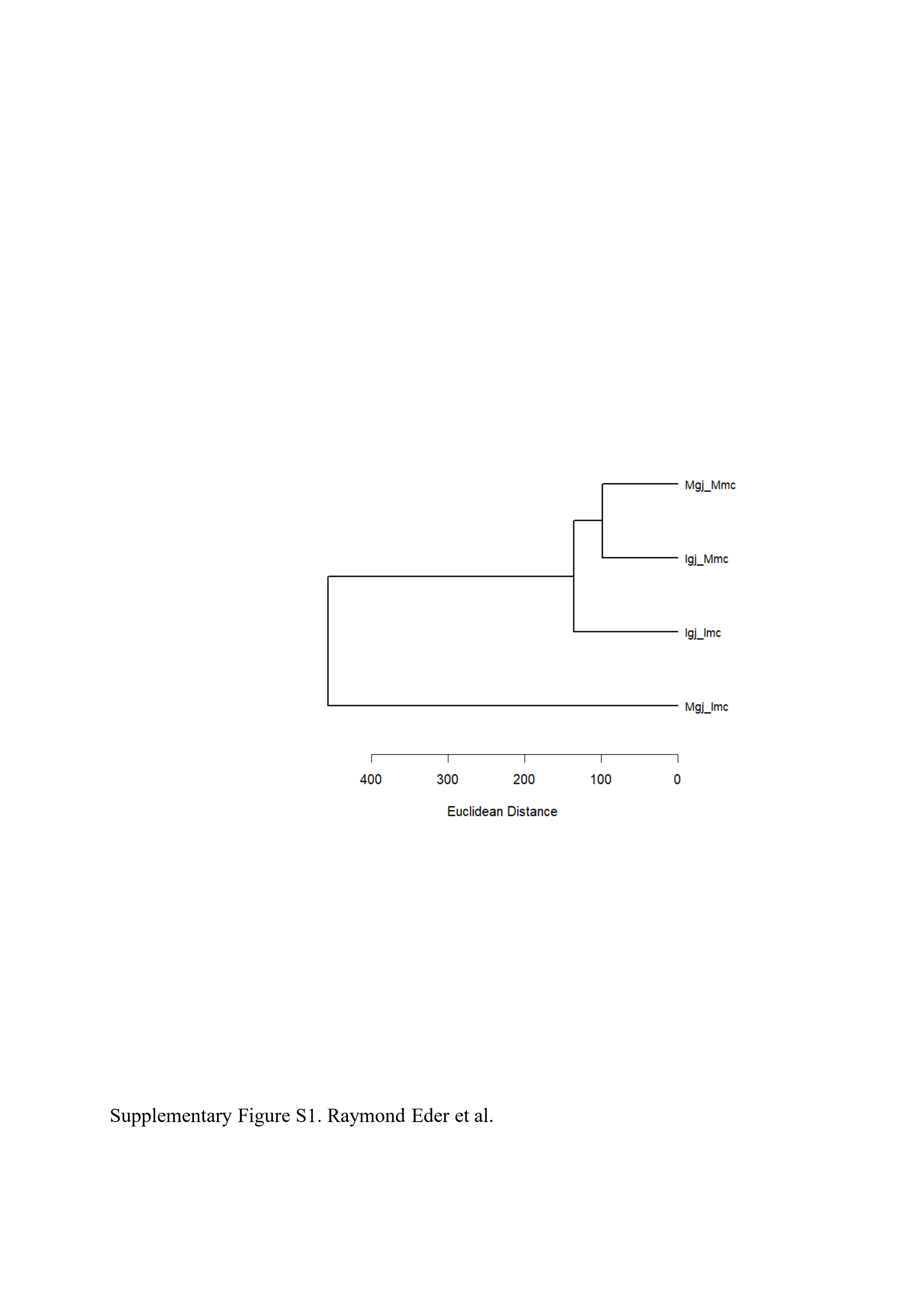

Supplement: Supplementary Figure 1 — Dendrogram resulting from hierarchical cluster analysis based on Euclidean distances calculated from the average profiles of volatile compounds in each fermenting condition (i.e., Igj/Imc, Igj/Mmc, Mgj/Mmc, and Mgj/Imc) at 96 hours ( Supplementary Table S3 ). Each point of the dendrogram represents the centroid of three biological replicates performed in the study. [file Image1.jpeg]

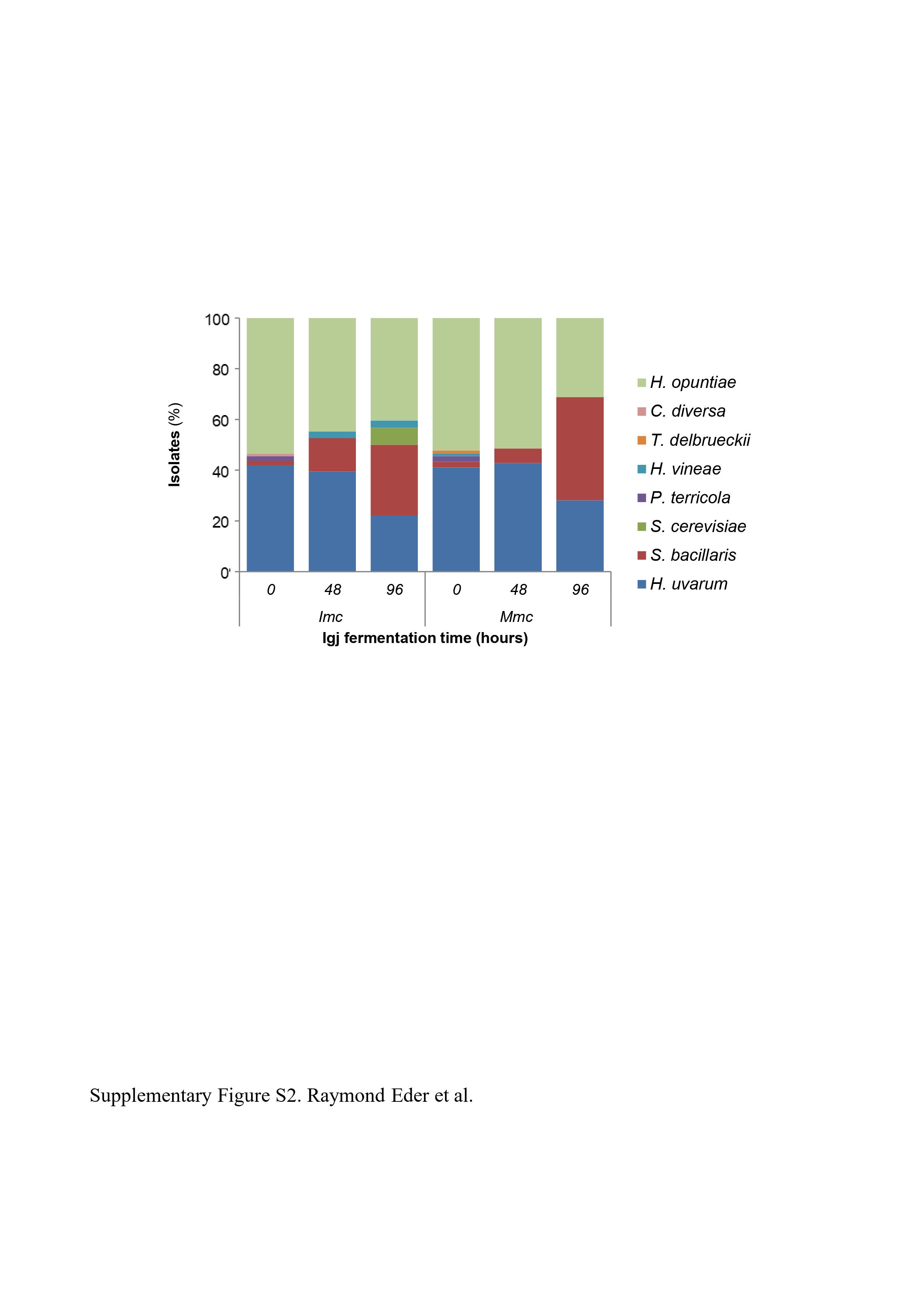

Supplement: Supplementary Figure 2 — Main represented yeast species at the initial stages of fermentations (0 to 96 h) of Isabella grape juice using Isabella (Imc) or Malbec (Mmc) microbial communities as inocula. Percentages represent the relative contribution of the indicated yeast species among the colonies obtained at the indicated times of fermentation. [file Image2.jpeg]
